# Evaluation of the advanced air mobility potential for organ transplantation in Austria and Germany

**DOI:** 10.1038/s41598-024-81045-2

**Published:** 2024-11-30

**Authors:** Robin Karpstein, Jakob Brolli, Philipp Stiegler, Robert Sucher, Florian Holzapfel, Peter Biberthaler

**Affiliations:** 1 Technical University Munich, Institute of Flight System Dynamics, Garching, 85748 Germany; 2 Clinical Department of General, Visceral, and Transplant Surgery, Medical University Graz, Graz, 8036 Austria; 3 Institute of Trauma Surgery, Munich, Technical University Munich, Munich, 81675 Germany

**Keywords:** Aerospace engineering, Statistics, Scientific data, Health care, Medical research

## Abstract

Solid organ transplantation continues to be the only or most efficient therapeutic solution for several end-stage diseases. The success of such transplantation is largely dependent on the swift transportation of organs from donors to recipients, as Cold Ischemia Time (CIT) plays a critical role in determining the recipient’s medical outcome. This study explores the potential of Advanced Air Mobility (AAM) in the context of organ transplantation in Austria and Germany. AAM, in the healthcare sector, is associated with potential overall process time savings via air transportation, thereby reducing CIT. However, the application of AAM for organ transplantation has not yet been implemented in Europe. This study employs a Monte Carlo simulation to derive the trip length distributions for organ transplantation in Austria and Germany. By utilizing data from Eurotransplant (2018–2021) and ÖBIG (2017–2021), it was found that 48% of organ transports within Germany, and 80% of organ transports within Austria, fall within a trip length of less than 150 km. This distance is within the capabilities of today’s AAM technology. Anticipated time benefits of up to 30 min compared to ground-based transport can be expected. Furthermore, the optimization of the organ transport process, facilitated by AAM, promises greater potential for CIT reduction.

## Introduction

Solid organ transplantation, the only cure for several end-stage diseases, confronts numerous challenges in healthcare. Apart from organ shortage, technical and logistical issues arise during transplantation. One significant factor is time, along with the associated ischemic tissue damage during organ transport from donor to recipient^1,2^.

Current healthcare trends, such as labor shortage^3^ and the centralization and specialization of hospitals^4–8^, are impacting Germany and Austria. The full effects will become evident in the medium-term until 2030^9–11^. As a result of centralization, specialization, and labor shortage, health service offerings are decreasing across hospitals in Germany and Austria. In some instances, this trend is also accompanied by the closure of hospital sites. Consequently, the mobility demand for information, goods, and people increases. Research indicates that ground-based transportation times with cars have increased^12^, and ground-based transportation often lacks sufficient availability for organ transplantation. Helicopters could offer similar benefits to AAM; however, helicopters are scarce and expensive compared to the potential use of AAM. Furthermore, both ground-based transportation means and helicopters are dedicated to other use cases, such as emergency response. Therefore, innovative, novel mobility options should be considered to maintain high-quality and needs-based healthcare. Additionally, battery-powered AAM aircraft help to reduce local emissions. Hence, AAM is a potential solution for goods and people transport in healthcare^13,14^.

AAM refers to novel aircraft concepts such as unmanned aerial vehicles (UAVs) and electrical vertical take-off and landing (eVTOL) aircraft (e.g., air taxis), as well as use cases like Urban or Regional Air Mobility^15^. ADAC, in collaboration with Volocopter^16^, and Nakamoto^17^ investigated the use of eVTOL aircraft for doctor shuttles in emergency response, and Goyal^18^ discussed the benefits of eVTOLs in emergency response more broadly. However, the more promising short-term application of AAM technology in healthcare seems to be UAVs, often referred to as drones in everyday language. Mesar^19^ proposes to use drones for the delivery of medical supplies during medical care in the field, while Handford^20^ and Pickell^21^ make a case for military medical evacuation, and Ling^22^ proposes drones for blood delivery use cases, which Nisingizwe^23^ applied in Rwanda and found a time benefit and reduced blood product wastage. In Germany, Baumgarten^14^ summarizes ongoing projects for the same aforementioned use cases and expands it by proposing it for rapid laboratory testing of frozen sections. Baumgarten^14^ highlight that the major challenges today are technological, e.g., providing sufficient range, and regulatory, e.g., providing a framework for certification and operation of drones in (un-)controlled airspace beyond visual line of sight. In the context of organ transplantation, the requirements on AAM and thus the size of the AAM vehicle depend on whether frozen sections and other samples, the organ itself, the organ in a perfusion machine, or the organ chaperoned by the operating team are transported, as well as the distance of the organ transport. In the first two cases, the size is comparable to today’s UAVs used in the cases described before^14,20,22,23^, while in the last case that involves people transport, eVTOL aircraft comparable in size to today’s emergency rescue helicopters are required.

Given the time-sensitive nature of organ transplantation and the inherent unpredictability, forward-looking optimization of resource planning for organ transports is unfeasible. Consequently, the benefits from improved planning are limited, thereby shifting the focus to the organ transport itself and the overall transplantation process. AAM has the potential to significantly enhance organ transports as current mobility options are not entirely satisfactory due to their availability, speed, and triage with conflicting use cases requiring the same transport resources. Sage and Scalea^24–26^ have demonstrated the medical feasibility of organ transports by drone within cities in North America. They underscore its immense potential and the need for further technological advancements, as well as understanding the aircraft requirements to expand beyond intra-city use cases, as the larger potential lies in city-to-city organ transports covering longer distances.

This study analyzes the range requirements for organ-transporting AAM aircraft in Austria and Germany to enable hospital-to-hospital organ transport and discusses the implications and benefits on the organ transplantation process. Austria and Germany are part of Eurotransplant, a European organization that facilitates the allocation and cross-border exchange of deceased donor organs between eight member countries: Belgium, Netherlands, Germany, Luxembourg, Austria, Hungary, Slovenia, and Croatia. The matchmaking at Eurotransplant is based on donor-recipient matching, taking into account medical and ethical criteria. In the process, the national organ balance between countries and the time on the waiting list are also considered.

## Results

The results of this study, simulating the trip length distributions for organ transplantation in Austria and Germany by utilizing historical data from Eurotransplant (2018–2021) and ÖBIG (2017–2021), are presented as cumulative distribution functions, comparing the weighted Monte Carlo simulation results with an unweighted distribution (Table 1). The weighted Monte Carlo results provide a more accurate representation of reality, as they incorporate historical data on organ transport. In contrast, the unweighted distribution assumes that each organ is transported exactly once between each possible origin and destination pair, without considering historical transport patterns. In Germany, the simulated distribution has a median of 159 km and a mean of 200 km, while the obtained distribution for Austria shows a median of 67 km and a mean of 90km. Thus, both distributions are right-skewed. Comparing the simulation results with the unweighted trip length distribution, i.e., assuming each organ is transported exactly once on all possible routes the organ can take (uniform allocation to possible routes), the right-skewness, i.e., the tendency towards shorter trips compared with all possible trips, is confirmed for both Germany and Austria. The German unweighted distribution has a median of 293 km and a mean of 298 km. The Austrian unweighted distribution has a median of 167 km and a mean of 199 km. Given the areal expansion of Germany and Austria, it is unsurprising that the maximum trip length is greater for Germany (826 km) than for Austria (518 km). Table 1 depicts both distribution statistics.

**Table 1:** Median and mean of the Monte Carlo (MC) Simulation and the unweighted trip length distribution for Germany and Austria by organ.

Country	Organ	Median, km		Mean, km	
		Weighted MC	Unweighted	Weighted MC	Unweighted
Germany	All	159	293	200	298
	Heart	287	293	292	299
	Lung	288	280	300	290
	Liver	220	293	248	300
	Kidney	111	291	141	297
	Pancreas	148	300	187	303
Austria	All	67	167	90	199
	Heart	97	210	108	214
	Lung	109	210	113	214
	Liver	103	165	102	199
	Kidney	58	156	73	185

In Table 2, the share of trips addressable up to a given trip length is depicted. The share of trips up to a certain length is essential in determining the potential of AAM for organ transplantation, as this directly defines the range requirements for AAM aircraft. Most AAM aircraft currently utilize battery-electric powertrains, with current battery technology being the primary factor influencing range limitations. Thus, the first key metric in assessing AAM potential is the range requirement. The dominant range cut-offs for current technology are approximately 50, 100, and 150 km. Most AAM aircraft today have ranges between 50 and 150 km (e.g., Wingcopter, Zipline, Rigitech), with a select few targeting ranges of up to 200 km (e.g., Joby, Lilium). Thus, 150 km is chosen as the critical range for evaluating the AAM potential in organ transplantation. The results show that 48% of German transplantations and 80% of Austrian transplantations are within a trip length of 150 km.

**Table 2:** Share of trips below specific distances for Germany and Austria on organ level.

Country	Organ	Share of trips, %			
		<50 km	<100 km	<150 km	<200 km
Germany	All	13	32	48	50
	Heart	5	12	20	31
	Lung	4	11	20	31
	Liver	8	21	34	46
	Kidney	18	45	64	78
	Pancreas	14	35	50	64
Austria	All	41	58	80	89
	Heart	33	51	73	83
	Lung	34	47	65	80
	Liver	33	48	75	87
	Kidney	48	67	88	93

Due to the organ’s different acceptable Cold Ischemia Time (CIT) limits and varying transport procedures, e.g., heart and lung transplantation are mostly accompanied by the operating team, while for kidneys and livers this is seldom the case, it is worth analyzing the data on an organ level for each country to uncover further insights.

### Germany

**Fig. 1:**
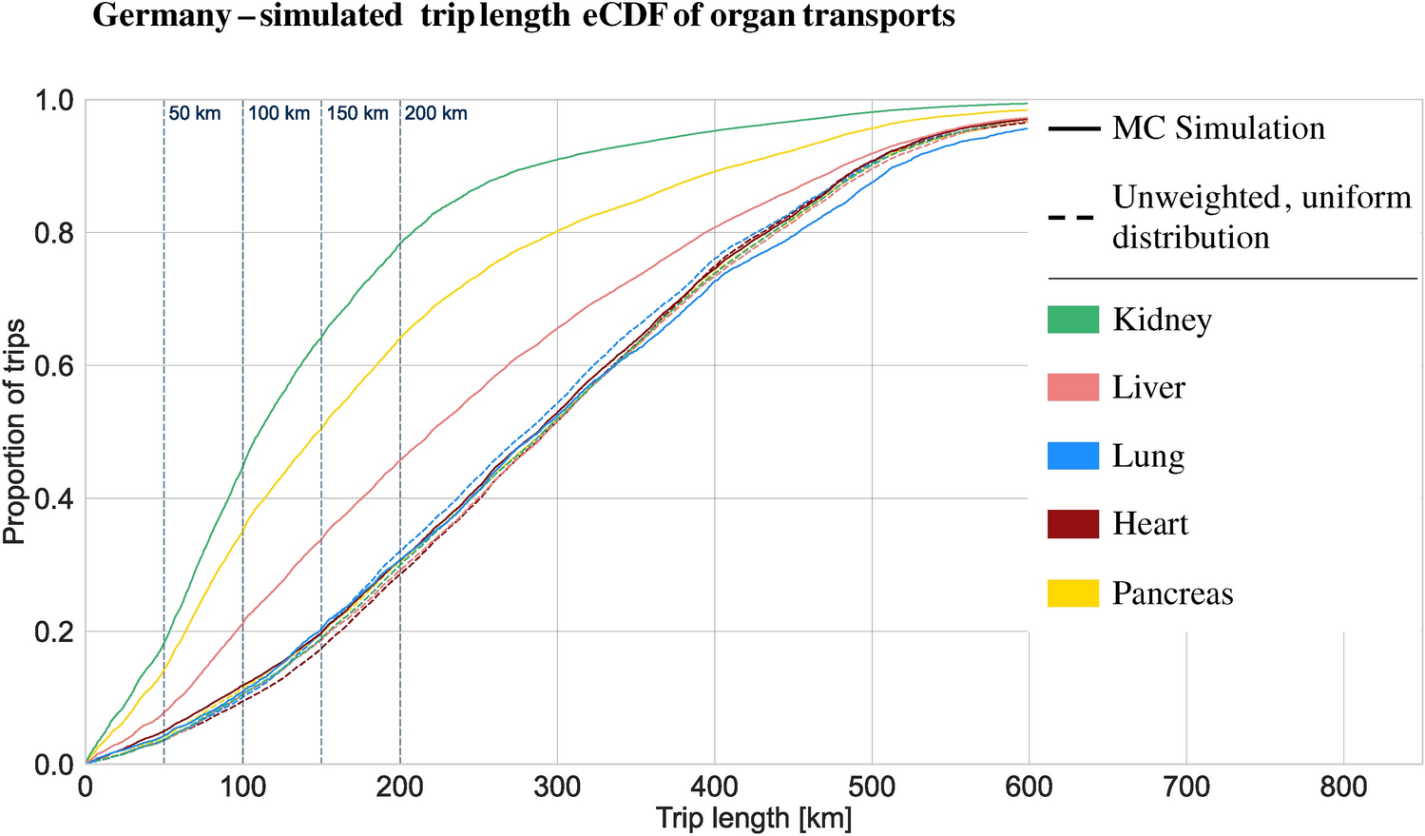
Normalized trip length simulated empirical cumulative distribution function for Organ Tansplantation in Germany. Based on weighted Monte Carlo Simulation with 10,000 iterations and Eurotransplant data from 2018 to 2021 (included).

The simulated trip length cumulative distribution for Germany in Fig. 1 shows that heart (median: 287 km, mean: 292 km) and lung (median: 288 km, mean: 290 km) transplantation transports are similar to a normal distribution and in line with the unweighted theoretical distribution. Meanwhile, liver (median: 220km, mean: 248km), kidney (median: 111 km, mean: 141 km), and pancreas (median: 148 km, mean: 187 km) transplantation are clearly right-skewed. Liver, kidney, and pancreas organ transport distributions also show the tendency towards shorter distances compared with the theoretical distribution.

### Austria

**Fig. 2:**
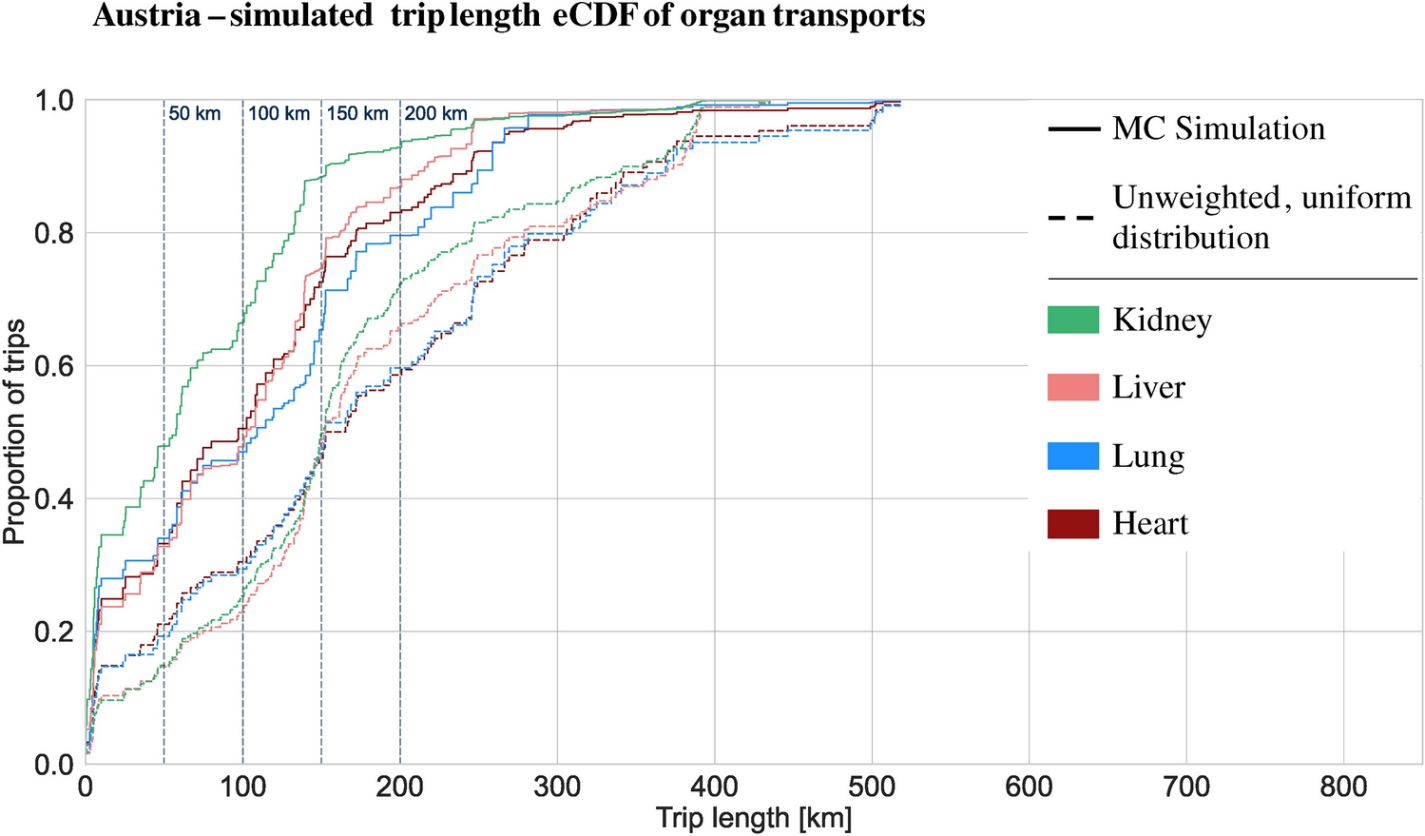
Normalized trip length simulated empirical cumulative distribution function for Organ Tansplantation in Austria. Based on weighted Monte Carlo Simulation with 10,000 iterations and ÖBIG data from 2017 to 2021 (included).

The simulated Austrian organ transplantation’s trip length cumulative distribution depicted in Fig. 2 shows a slightly different behavior compared to the German transplantations. Heart transplantations (median: 97 km, mean: 108 km) show greater right-skewness compared to the German data, while lung (median: 109 km, mean: 113 km) and liver transplantations (median: 103 km, mean: 102 km) appear to be unskewed when considering the median and mean. While kidney transplantations are the only clear right-skewed distribution (median: 58 km, mean: 73 km), all simulated organ transport trip lengths are on average shorter than the unweighted distribution as indicated by the median and mean of the Monte Carlo simulation and the unweighted distribution in Table 1 and shown in Fig. 2.

As a summary of the simulated distributions and as an indication for required ranges of AAM aircraft and potentially addressable trip shares, Table 2 depicts the share of trips below 50 km, 100 km, 150 km, and 200 km. Across Germany, 48% of trips are within a distance of 150 km. On organ level, this is 20% of heart and 20% lung transplantations, 34% of liver transplantations, 64% of kidney, and 50% of pancreas transplantations. In Austria, a higher share of trips are rather short and addressable as overall 80% of trips are within 150km. 73% of heart transplantations are within a 150 km distance, 65% of lung transplantations, 75% of liver transplantations, and 88% of all kidney transplantations.

### Time benefit estimation

The time benefit estimation dataset contains 166 liver transplantations in Graz from 2017 to 2021, matching the exact number in the OEBIG dataset (cf.^27^). CIT is available for all transplantations, while transplantation surgery duration data is available for 116 cases. The mean transplantation duration is 2.5 h, with a median of 2.0 h a lower quartile of 1.95 h, and an upper quartile of 2.16 h. Therefore, a transplantation duration of 2.0 h (120 min) is assumed. For duration calculations, the values as specified in the “Methods” section are used. We excluded three entries where our conservative time assumptions would result in no logistics time, making them irrelevant for the analysis. Additionally, we retained only those entries where the resulting average travel speed was between 75 and 150 kilometers per hour, which is considered a reasonable average speed for organ transport by car. This filtering process left 17 entries for analysis. For the lab-transporting vehicle, a mean time saving of 21.5 min is observed and a median time saving of 19.2 min (Standard Deviation: 27.9 min, IQR: 41.0 min). For the organ-transporting vehicle, the mean time saving is 36.9 min, with a median time saving of 35.1 min (Standard Deviation: 31.3 min, IQR: 68.7 min). For the medical personnel-transporting vehicle, the mean time saving is 77.6 min, with a median time saving of 76.9 min (Standard Deviation: 42.1 min, IQR: 142.6 min).

## Discussion

In addition to organ shortage, technical and logistical issues during transplantation, a major factor is time and the associated ischemic tissue damage during organ transport from donor to recipient^28,29^. The benefits of CIT reduction , one of the few things we can modify for any potential and specific organ donor, are proven and widely accepted^30–35^. Organ transplantation is a critical component of clinical care worldwide, with liver, kidney, heart, lung, pancreas, and intestine transplantation being routinely performed, and both patient and allograft survival continuing to improve^36^. The success of transplantation relies on various factors, including organ preservation and minimizing ischemic tissue damage during transport. The current transportation process faces challenges related to time, logistics, and technical issues. AAM technology, which has not yet been implemented in Europe for organ transports holds significant potential in addressing these challenges and optimizing the transplantation process as the transport distances of organs allow AAM aircraft to cover a substantial share. AAM aircraft are a viable option in the near to medium-term future as, even today, a substantial portion of organs are used to air transport by using helicopters and private or commercial aircraft. In the transport chain, there is often an additional ground-based segment to cover the trip from the hospital to the airport or heliport, especially when no landing site is available directly at the hospital. Regions with better connectivity to airports and heliports thus might have more transport options and, consequently, better CIT results. In the future, AAM aircraft could enhance connectivity for regions with limited airport access, offering a lower-cost alternative to helicopters. In the studied countries, Germany and Austria, substantial potential exists since each Austrian hospital has a heliport, all transplant centers in Germany are equipped with one, and nearly all German organ procurement centers have a landing site at or near their location. We explore and discuss the potential benefits of AAM in organ transplantation, including reduced CIT and overall process optimization.

The preservation of organs is a crucial aspect of the donation and transplantation process^37^. Ongoing research aims to improve transplantation outcomes through strategies such as machine perfusion and supplementation of preservation solutions, which are partly incorporated into the clinical routine^38–42^. However, apart from organ shortage, time and ischemic tissue damage during transportation pose significant challenges^28,29^. The reduction of CIT has been widely discussed and accepted in research due to its impact on graft loss risk^30–35^. Strategies to address these challenges are essential for improving transplantation outcomes. In our study, we estimated the organ transport ranges using a Monte Carlo simulation because the data on the Eurotransplant region was not available.

Our limited time benefit estimation indicated that there is potential for AAM vehicle to replace car-based transport in the organ transplantation context, particularly as AAM vehicle speed increases. The results obtained from this study are applicable to other car-based transports in the context of organ transplantation. In the “Methods” section, we introduced three possible AAM applications from our perspective: The transport of lab samples in the context of organ transplantations (e.g., frozen section, blood), the organ itself (plus lab samples), and the medical team (plus the organ itself and lab samples). The rationale for this structure is the availability of AAM vehicles capable of carrying the required payload. There are vehicles capable of transporting only lab samples. The next step up in payload is the organ itself, together with the lab samples. The largest relevant AAM vehicle category (not yet certified) is able to transport the medical team with the organ and the lab sample. If we introduce these vehicles in the organ transplantation context today, the overall process and organization through Eurotransplant and the coordination offices will likely remain the same initially. This means that the coordination offices send the retrieval teams to the retrieval hospital. The retrieved organ and lab samples either travel with the medical team back to their transplant center if it is allocated there, or the organ and lab samples travel independently to their specified destination transplant center. Depending on the regulatory approvals and especially technology availability, the most likely stepwise introduction process is to first test AAM technology with lab samples, then use it to transport organs, and finally, when the man-carrying AAM vehicles are proven and available to supplement the helicopter fleets, they should be introduced to healthcare.

Regarding lab samples, the potential time savings could exceed the observed mean of 21.5 min if the samples are transported independently of the organ. Currently, organs are received at the hospital in Graz, and upon arrival, the frozen section and the donor’s blood are taken to the laboratory for testing. This process results in unnecessary delays due to the time required to perform the laboratory analyses and subsequently prepare the recipient for transplantation. Samples can be shipped immediately after retrieval at the beginning of the organ procurement procedure, potentially reducing CIT by 60 min or more. This enables faster determination of the donor-recipient match, early recipient preparation, and immediate transplantation upon organ arrival. Additionally, in cases of unacceptable match results, the organ can be offered to other patients earlier, saving valuable time. However, a caveat of this approach is that a second transport would be required for the medical team if they are traveling to the same destination as the organ. For pure organ transport, the benefits extend beyond substantial time savings. There is also potential to increase flexibility in the logistics process and reduce dependence on human transport personnel. The greatest absolute time savings are achieved when transporting the medical team using AAM vehicles instead of ground-based methods. The challenge in this scenario is that AAM manufacturers must still validate their claims that these aircraft can be operated at costs comparable to taxi services. In summary, we believe the greatest potential lies in the independent transport of lab samples separate from the organ, organ transport when the medical team is not traveling to the same destination, and the transport of the entire medical team with or without the organ. The potential benefits of CIT reduction through AAM are substantial, as every hour of reduction translates to a 3.4% decrease in liver graft loss risk according to Lozanovski^30^. The analysis conducted highlights the need to also track the logistics process and additional timestamps (e.g., departure and arrivals, internal logistics duration) to enable a holistic data-driven assessment and impact estimation of AAM technology or other potential changes to the logistics process.

In addition to reducing transport time through faster transportation methods, there are other strategies to minimize CIT. One approach is to prioritize local recipients, thereby reducing the geographical distance between the donor and recipient, which should directly lower CIT. Eurotransplant, the organization in which this study is situated, already implements this strategy unless the case is classified as high urgency. Additionally, optimizing internal hospital processes to facilitate “just-in-sequence” surgeries could further reduce CIT. Another potential approach is the transportation of either the donor or recipient to minimize CIT. However, transporting the organ donor presents significant logistical and medical challenges, as well as ethical and legal concerns. Conversely, the transport of organ recipients occurs infrequently.

The clinicians’ behavior in organ allocation could be affected if a CIT reduction can be proven in practice by applying AAM technology. By substantially accelerating the transport process, clinicians might decide to allocate organs to farther distant recipients, which counteracts the positive effect. However, transporting organs farther could also allow for a better donor-recipient fit or cover high urgency recipients better, hence, the longer CIT might not affect the medical outcome negatively. Furthermore, machine preservation and hypercooling techniques might reduce the necessity to focus on CIT, hence, currently existing allocation algorithms need to be revisited.

Advancements in machine perfusion are expected to significantly enhance transplantation success. This technology enables a thorough evaluation of less ideal organs, such as those from circulatory death donors or extended criteria donors, thereby expanding the pool of viable organs. In the future, genetic modifications and medical treatments applied during perfusion could become feasible, which may reduce the need to minimize logistical transport time^38,43^. The objective of employing Advanced Air Mobility (AAM) vehicles is not just to reduce transport time but also to increase the flexibility of the logistics process and reduce dependence on transport personnel. Future AAM vehicles are anticipated to have the capability to transport perfusion machines, effectively combining two process steps into one streamlined process.

The transport process of organs, including factors such as time and distance, is not the sole determinant of CIT. Other important factors include the duration of organ procurement and transplantation procedures, internal logistics within the hospital, and waiting times for laboratory test results that assess the organ’s transplantability. However, the transport process is the only part AAM technology might be able to influence. This study uses the range of organ transportation as a proxy to estimate the proportion of organ transplantations that could potentially be supported by Advanced Air Mobility (AAM) technology. The rationale for this approach lies in the current limitations of AAM vehicles, primarily due to existing battery technology. As battery technology advances and fuel-cell-based powertrains become available, other, yet-to-be-identified factors may emerge as more suitable proxies. Additionally, every hospital in Austria, all transplant centers in Germany, and many organ procurement hospitals in Germany have at least one helipad. This infrastructure ensures direct access to air transport, eliminating the need for additional ground travel between airports and hospitals. This setup has the highest potential for routes up to 150-200 km, where automobiles are predominantly used today, aligning well with the capabilities of current AAM technology.

This study, focusing on Germany and Austria, reveals that 48% of German and 80% of Austrian organ transportation trips have a trip length of less than 150km, making them addressable by AAM in the short term. The distribution of trip lengths shows a skew towards shorter distances compared to the unweighted distribution, indicating the potential for efficient AAM application.

The unweighted distribution assumes a uniform distribution of origin and destination pairs, i.e., each organ is transported exactly once from each possible retrieval hospital to each possible transplant center. This assumption (obviously) does not reflect reality, but would be a good first estimate without additional data and information. The weighted Monte Carlo simulation result based on historical data confirms that a uniform unweighted distribution does not reflect reality and should not be used. Additionally, using it as a reference would lead to the (wrong) conclusion that in the short-term AAM technology appears unpromising as the range is substantially exceeded. Therefore, the unweighted uniform distribution serves purely as a baseline for comparison and underlines the necessity to conduct such analyses with real-world data. Eurotransplant’s allocation algorithm, which prioritizes for donor-recipient matching, taking into account medical and ethical criteria , while considering national organ exchange balance and logistical considerations, also aligns well with the simulation results. With Eurotransplant’s statistics library^44^, we can confirm higher urgency shares for hearts (Germany: 78%, Austria: 56%), lungs (Germany: 15%, Austria: 42%), and to some extent livers (Germany: 15%, Austria: 10%). The study also underscores the importance of shorter trips in Austria due to limited organ procurement and transplantation hospitals and the country’s mountainous geography.

AAM technology has demonstrated significant potential in healthcare^14,19,22,23,45,46^, including time-critical organ transplantation^24–26^. By reducing transport time, AAM can effectively reduce CIT. The range of possible destinations also expands, enabling better allocation of organs. Scalea and Sage^24–26^ used a small unmanned drone and argue that while inner-city transports show potential, transports from city-to-city or intra-regional transport by drone have even more potential and benefits. Our study assessed the potential for Germany and Austria to conduct organ transports using AAM. We find that 48% of German and 80% of Austrian transplantations fall within a trip length of 150km, making them suitable for AAM. Battery-driven drones already achieve practical ranges of around 100km, while future developments and improving battery technology will enable ranges exceeding 150 km by 2030.

Different aircraft types serve specific purposes in organ transportation. Air taxis, such as the Lilium Jet and Joby’s S4 aircraft, are suitable for transporting heart and lung transplants, as they offer higher payload capacity and can accommodate the operating team that usually chaperones the organ until successful transplantation. Unmanned drones are ideal for transporting organs like the liver, kidney, and pancreas between hospitals. In cases where the distance between hospitals exceeds drone range capabilities, reducing transport time from the hospital to the airport becomes crucial. By utilizing both aircraft types, the transportation of a significant percentage of organs in Germany and Austria can be drastically improved. This improvement enhances allograft quality, acceptance, and ultimately improves the quality of life and lifespan of recipients. Additionally, the adoption of AAM frees up capacity for medical and emergency personnel by eliminating the need for their transportation vehicles.

While the model developed in this study provides valuable insights, the exact origins and destinations of organ transportation could not be determined due to the unpredictable nature of organ donors’ deaths. Pseudo-randomized simulations based on regional origin and destination data were used to derive initial range requirements for AAM vehicles. However, the data lacked information on transport means and transport time, which would have quantified the logistical benefits of AAM more accurately. The German data includes living kidney donations, which do not involve transportation. Eurotransplant was unable to exclude or specifically identify living donations in the data, so we couldn’t filter them out ourselves. In contrast, the Austrian data does not include living donations, as they are excluded by OEBIG.

In conclusion, this study provides valuable insights into the potential application of AAM in organ transplantation in Germany and Austria. This novel technology system of organ transport could reduce the burden on the healthcare system and transport capacities. The findings suggest that a significant percentage of organ transports in these countries could be improved with the use of AAM, leading to shorter ishemia times with enhanced allograft quality, acceptance, and ultimately, improved quality of life and lifespan of recipients. However, further research is needed to address the challenges related to UAV technology scalability, airspace integration^47–49^, safety and regulation, and infrastructure. The infrastructure requirements for AAM at hospitals can be easily met, as existing helicopter landing sites are available at most hospitals with emergency departments and organ transplantation activities. With careful planning and coordination, AAM could revolutionize the field of organ transplantation, offering a more efficient and effective solution to the logistical challenges currently faced in the process.

## Methods

The analysis is grounded on historical Eurotransplant and ÖBIG organ transplantation data, as well as German and Austrian hospital databases. A Monte Carlo simulation is employed to simulate the trip length distribution of organ transports. In addition to the trip length distribution, the share of trips given a specific trip length is of interest to estimate the technical feasibility with today’s AAM technology and anticipated future developments until 2030.

### Organ transplantation data

Organ transplantation data encompasses implicit allocation decisions (e.g., urgency) and inherent coincidence of the location of the (deceased) donors that are not modeled in this study. By utilizing historical data, the study aims to account for these factors.

#### Eurotransplant data from 2018-2021 for Germany

Eurotransplant provided organ transplantation data from 2018 to 2021 (inclusive) for heart, lung, kidney, liver, and pancreas transplantation. The data includes the origin and destination region according to the German Organ Procurement Organization (DSO) and indicates whether the whole organ or parts thereof are transplanted. For this study, parts of an organ are assigned to the entire organ category because for the transport it’s irrelevant whether parts of or the entire organ is transported. In total, there were 15,483 organ transplantations (heart, lung, liver, kidney, pancreas) in Germany from 2018 to 2021, of which 1,673 (10.8%) were transported to another country and the remaining 13,810 (89.2%) organs were transplanted in Germany. Since the destination country of donations to other countries than Germany is unknown, these 1673 donations are discarded from the data for this study. Of the remaining 13,810 organ donations, there are 7467 (54.1%) kidney, 2906 (21.0%) liver, 2077 (15.0%) lung, 1058 (7.7%) heart, and 302 (2.2%) pancreas transplantations.

#### ÖBIG transplantation data from 2017-2021 for Austria

The Austrian organ transplantation data is sourced from the annual report of ÖBIG^27^ as data from Eurotransplant is unavailable. To receive Austrian data through Eurotransplant, permission by each individual Austrian hospital is required. The report contains organ transplantation data for the heart, lung, liver, and kidney from 2017 to 2021. The data includes the donor region and the recipient’s transplantation center, which is used as an indicator for the origin and destination of the organ transport. This study focuses on the intra-Austrian transport, excluding the province of Bozen. The data consists of 2914 organ transplantations, of which 1486 (51.0%) are kidney, 770 (24.8%) liver, 401 (13.8%) lung, and 305 (10.5%) heart transplantations.

### Origin and destination hospitals

The precise locations of the origin and destination hospitals are crucial to determine the distance between two hospitals. Python’s geopy package with OpenStreetMap is used to query the GPS location of each hospital based on its street address. The German organ procurement hospitals are available on the DSO website^50^, and after data cleansing, a final list of 1162 organ procurement hospitals is obtained. German transplantation centers are also provided by DSO^51^. In total, there are 47 unique transplantation center locations, each with a distinct transplantation program: 20 perform heart transplantation, 13 lung transplantation, 20 liver transplantation, 38 kidney transplantation, and 26 pancreas transplantation. All Austrian hospitals are listed by the Austrian health ministry in a searchable manner^52^. In total, there are 164 unique hospital locations. According to ÖBIG^27^, 73 perform organ donations and retrieve the organ themselves, thus representing the origin of the organ transport. Austrian transplantation centers are in Graz, Vienna, Innsbruck, and Linz. Graz, Innsbruck, and Vienna perform all solid organ transplantation, while Linz performs kidney transplantation only.

### Monte Carlo simulation

Due to GDPR considerations, access to the historical origin and destination hospitals corresponding to the organ transplantation data provided by Eurotransplant and ÖBIG is unavailable. Therefore, a Monte Carlo simulation is used to probabilistically approximate the trip length distribution of organ transports between the hospitals participating in organ transplantation. Pseudorandomized samples are suited to simulate distance distributions of organ transplantation given the inherent randomness of organ transplantation that (with the exception of living kidney donors) happen randomly when a person dies. The random seed is set to 19, and the origin-destination hospital combination is pseudorandomly sampled from all possible origin-destination hospital combinations for a given organ and origin - destination region pair. The sample size is determined based on the historical occurrence of organ transports between origin and destination regions, in essence, this is an empirical cumulative distribution function. For each origin-destination hospital combination, the great circle distance between the hospitals is calculated and stored together with the sample mean and median. In total, this procedure is repeated 10,000 times per organ origin region—destination region combination, which represents a simulation of 40,000 years in the case of Germany and 50,000 years in the case of Austria. .

#### Germany

The German transplantation data is available on DSO regional level. This means organ procurement and transplantation hospitals are assigned to one DSO region. Given the origin region, destination region, and transplanted organ, there is a subset of possible origin-destination hospitals from which the sample is drawn.

#### Austria

In Austria, this varies as the organ origin is known on the country state level (e.g., *Styria*), while the destination is known on the hospital level due to the fact that there are only four transplantation centers in Austria and ÖBIG publishes this information. Hence, the origin hospitals are sampled from the subset of hospitals in each region, while the destination hospital is directly identified without sampling.

### Time benefit estimation

As mentioned above, logistics duration is not consistently tracked in the Eurotransplant data and therefore is unavailable. To assess potential benefits, the logistics time is an important metrics and must be estimated using the limited data available. The analysis focuses on liver transplantation data from Austria between 2017 and 2021, specifically involving cases where the transplant coordination center in Graz was part of the process. The primary reason for this focus is that maintaining a low CIT is critical for liver transplants, necessitating swift and efficient procedures without unnecessary delays. Additionally, this dataset is advantageous because it is our own, allowing us to fully understand its collection methods and any inherent biases or errors, which would be uniformly present across the entire dataset. The CIT is consistently tracked and serves as the foundation for our estimations.

CIT is composed of the following time intervals: Procurement, packaging, and transport to the hospital entrance; Logistics (e.g., car, helicopter, aircraft, or a combination); Internal logistics to the operating room; Organ assessment and biopsy; Organ preparation, concurrently with recipient preparation; Transplantation until successful re-establishment of blood flow. Based on our available data and practical experience, we estimate the duration of each step as follows:

1. Procurement, packaging, and transport to the entrance: Based on experience and observations from three organ procurements in 2024, this step consistently takes between 90 and 120 min. The conservative value of 120 min is taken.
2. Logistics Duration: This is the target metric, calculated by subtracting the durations of steps (1) and (3–6) from the tracked CIT.
3. Internal logistics to the operating room: Typically takes 15–20 min based on experience at our facilities. The conservative estimate of 20 min is chosen.
4. Organ assessment and biopsy: This step is primarily determined by the biopsy duration, which typically takes around 60 min.
5. Organ preparation and recipient preparation: On average, this process takes 60 min.
6. Transplantation until re-establishment of blood flow: According to our data, this step averages 2 h.

We only include data from cases without machine perfusion, where organs were not transported by plane, and where the procurement center was in Austria but not in Graz. The time benefit is then calculated based on the expected flight time, determined by multiplying the distance between origin and destination with the average cruising speed of the potential vehicles.

For transporting laboratory samples, suitable vehicles include the Rigitech Eiger (https://rigi.tech/wp-content/uploads/2023/09/Eiger-Technical-Spec-Sheet-Aug-2023.pdf, last accessed September 9, 2024) and the Striek Engineering CarryAir (https://www.striekair.com/_files/ugd/3dd05b_cabb329496ba4ea5b98412b05f4a103a.pdf, last accessed September 9, 2024), both with a cruising speed of approximately 105 km/h and adequate cargo bays and payload capacity. For organ transport, the Dufour Aerospace Aero2 (https://www.dufour.aero/aero2) is a suitable candidate with an efficient cruising speed of 150 km/h. Human transport-capable aircraft include helicopter-sized aircraft such as the Lilium Jet (https://lilium.com/) or Joby Aviation (https://www.jobyaviation.com/), which have an estimated cruising speed of 250 km/h.

## Data Availability

The publicly available data from ÖBIG, DSO, and gesundheitsliste.at are cited in the references. I can provide the Eurotransplant data upon request, please reach out to me via e-mail (robin.karpstein@tum.de). Alternatively, the data can be requested from Eurotransplant directly: https://www.eurotransplant.org/statistics/data-requests/
